# Nanoliposomal Delivery of MicroRNA-203 Suppresses Migration of Triple-Negative Breast Cancer through Distinct Target Suppression

**DOI:** 10.3390/ncrna7030045

**Published:** 2021-07-27

**Authors:** Shuxuan Song, Kelsey S. Johnson, Henry Lujan, Sahar H. Pradhan, Christie M. Sayes, Joseph H. Taube

**Affiliations:** 1 Department of Biology, Baylor University, Waco, TX 76706, USA; Shuxuan_Song1@baylor.edu (S.S.); Kelsey_Johnson1@baylor.edu (K.S.J.); 2 Department of Environmental Science, Baylor University, Waco, TX 76706, USA; Henry_Lujan@baylor.edu (H.L.); sahar_pradhan@baylor.edu (S.H.P.); Christie_Sayes@baylor.edu (C.M.S.)

**Keywords:** microRNA-203, EMT, nanoliposome, nanoparticle, microRNA, breast cancer

## Abstract

Triple-negative breast cancers affect thousands of women in the United States and disproportionately drive mortality from breast cancer. MicroRNAs are small, non-coding RNAs that negatively regulate gene expression post-transcriptionally by inhibiting target mRNA translation or by promoting mRNA degradation. We have identified that miRNA-203, silenced by epithelial–mesenchymal transition (EMT), is a tumor suppressor and can promote differentiation of breast cancer stem cells. In this study, we tested the ability of liposomal delivery of miR-203 to reverse aspects of breast cancer pathogenesis using breast cancer and EMT cell lines. We show that translationally relevant methods for increasing miR-203 abundance within a target tissue affects cellular properties associated with cancer progression. While stable miR-203 expression suppresses LASP1 and survivin, nanoliposomal delivery suppresses BMI1, indicating that suppression of distinct mRNA target profiles can lead to loss of cancer cell migration.

## 1. Introduction

Breast cancer is the most commonly diagnosed cancer and the second-largest cause of cancer-related deaths in women in the United States [1]. A majority of breast cancers are carcinomas, and their histological stratification is based primarily on the expression of estrogen receptor (ER), progesterone receptor (PR), and human epidermal growth factor receptor 2 (HER2). Triple negative breast cancers (TNBCs) are defined through their lack of these receptors and are more difficult to manage clinically owing to the lack of protein targets, in contrast to tumors with hormone receptor or HER2 positive breast cancer, which are conventionally targeted with hormone inhibitors such as estrogen-response modulators and aromatase inhibitors or HER2-blocking antibodies, respectively [2]. Most breast cancer patients do not succumb to their primary tumor, but instead to metastases that become apparent after the primary lesion has been removed. For cells to contribute to metastases they must leave the primary site, enter the vasculature, survive in the blood, and then extravasate, and colonize secondary sites. Despite metastasis being a complex, multistep process, clinical and experimental evidence supports a role for epithelial–mesenchymal transition (EMT) in driving metastasis [3,4,5,6].

EMT is an intricate series of molecular and morphological changes that allows epithelial cells to decrease their inter-cellular adhesion and apical-basal polarity, and acquire a mesenchymal morphology and motile phenotype [7]. EMT is known to contribute to development during formation of the mesoderm and drive epithelial wound healing throughout life. When aberrantly activated under pathological conditions, carcinoma cells exploit EMT to facilitate dissociation from the primary tumor, initiating the metastatic cascade [8]. The cellular shift from epithelial phenotype toward mesenchymal state is mediated by key transcription factors. Major EMT-inducing transcription factors (EMT-TFs) include zinc-finger-binding transcription factors SNAI1 and SNAI2 [9], the basic helix-loop-helix (bHLH) factors TWIST1 [10], mesenchyme Forkhead transcription factors FOXC2 [11], and the zinc-finger E-box-binding homeobox factors ZEB1 and ZEB2 [12]. Recent studies utilizing genetic mouse models with conditional deletions showed that EMT transcription factors SNAI1 and TWIST1 promote breast cancer metastasis [4,5], while ZEB1 is a strong promoter of metastasis in a pancreatic cancer model [13]. These EMT-TFs repress genes associated with epithelial cells such as CDH1 (E-cadherin) and induce the expression of mesenchymal cell-associated genes, ultimately leading to the cellular hallmarks of EMT [14].

MicroRNAs (miRNAs) are a class of small, endogenous noncoding RNAs of 22–25 nucleotides in length that play important roles in regulating genes that facilitate normal development and in diseases [15]. miRNAs are transcribed from different genomic locations by RNA polymerase II enzyme as a long primary transcript (pri-miRNAs) and cleaved by Drosha (RNase III family) to yield the precursor miRNA (pre-miRNA) in the nucleus. After transfer into the cytoplasm, the pre-miRNA is further processed into a miRNA/miRNA duplex by Dicer. Then, the duplex unwinds and the mature single-strand miRNA is subsequently loaded into the RNA-induced silencing complex (RISC) to form an miRNA-induced silencing complex (miRISC) [16]. The miRISC pairs with its complementary target mRNA in the 3′-untranslated regions (3′-UTR), which controls gene expression through sequence-specific interactions with target mRNAs, causing degradation or translational repression [17]. miRNA-mediated regulation of basic biological processes including proliferation and apoptosis is a common feature in cancer, inflammation, cardiovascular diseases, and metabolic disorders, suggesting that miRNAs could serve as targets for therapeutic intervention [18].

Several microRNAs play key roles in EMT and can directly regulate the expression of EMT transcription factors [19]. The miR-200 superfamily of miRNAs (miR-200a and -141 and miR-200b, -200c, and -429 [20]) plays a central role in EMT of many epithelial cancers [21]. Overexpression of miR-200 inhibits the expression of ZEB1 and other targets, while ZEB1 can bind and transcriptionally inhibit the promoters of two miR-200 transcription units, thus forming a double negative feedback loop [22,23].

An additional regulator of EMT is microRNA-203. Not part of the miR-200 family, miR-203 is located at chromosome 14q32-33 and possesses a distinct seed sequence (5′-gugaaauguuuaggaccacuag-3′). miR-203 can function as a tumor suppressor in cancers ranging from chronic myeloid leukemia (CML) [24] to human glioblastoma [25], rhabdomyosarcoma [26], and prostate cancer [27]. In a developmental context, miR-203 triggers differentiation in multilayered epithelia and is known to promote differentiation of normal epidermal stem cells [28]. As with miR-200, suppressed miR-203 expression can be driven by ZEB1 [29]. Epigenetic regulatory mechanisms such as DNA methylation and histone modification can also silence miR-203 expression [30,31]. Indeed, cancer cell lines from a range of tissue types exhibit elevated miR-203 expression when treated with 5-azacytidine or histone deacetylase inhibitors [29,31]. miR-203 activity can also be suppressed by the lncRNA chromatin-associated RNA 10 (CAR10), which is induced by EMT and interacts with miR-30 and miR-203 [32].

Triple-negative breast cancer cell lines exhibit low expression of miR-203, demonstrated by the upregulation of multiple targets with potential roles in the pathogenesis of this disease. miR-203 has been described as a stemness-inhibiting microRNA [31] by suppressing factors such as the self-renewal factor BMI1 [33,34], which functions as part of the polycomb repressor complex 2 (PRC2), which has been shown to repress genes such as CDH1 [35]. Another target, BIRC5 (survivin), is a key anti-apoptotic protein involved in a variety of cancer types that can also promote cell proliferation, thereby affecting cancer progression [36]. The miR-203 target LIM and SH3 protein 1, LASP1, is a nucleo-cytoplasmic shuttle protein involved in migration, adhesion, proliferation, and cell cycle progression in a variety of cancers [37]. LASP1 was originally identified from cDNA libraries of metastatic breast cancer, and its corresponding protein is overexpressed in more than 50% of breast cancers [38]. Additional characterized targets include ATM kinase [39], TWIST1 [40], fibroblast growth factor 2 (FGF2) [41], and receptor of Wnt signaling pathway Frizzled (FZD)-2 [42].

Developments in engineered nanomaterials, such as nanocrystalline drugs or nano-enabled drug carriers, are relevant to cancer in the fields of drug delivery, diagnosis, imaging, synthetic vaccine development, and miniature medical devices [43]. Key features including improved circulation and minimal systemic toxicity have shown promising applications for clinical development [43]. Common moieties include liposomes, polymeric micelles, and dendrimers. Based on each unique nanomaterial design, these nanoparticles offer extensive possibilities for cancer treatment.

In addition to the type of nanomaterials, the pharmacokinetics and biological distribution of nanoparticles are also related to the size, shape, and surface properties of nanomaterials [44]. Liposomes are closed spherical vesicles consisting of a lipid bilayer that encapsulates an aqueous phase in which drugs can be isolated, stored, and delivered [45]. The size of spherical lipid vesicles can range from a few nanometers to several micrometers, and nanoliposomes applied to medical use generally range between 50 and 450 nm [46]. Nanoliposomes are considered as an ideal drug delivery system because of their excellent ability to encapsulate many kinds of drugs similar to cell membranes.

Nanoliposomes can be preferentially accumulated in tumors, improve the efficiency of anticancer drugs, and reduce their systemic side effects, depending on the enhanced permeability and retention effect (EPR) [47]. Through the design of very small liposomes (<100 nm), tumor types with EPR can be utilized to realize the organization of the passive intake, thereby increasing drugs in the accumulation of specific targets [48]. Passive diffusion takes advantage of the EPR effect while also reducing toxicity, including inflammatory response, caused by reduced blood circulation to off-target areas of the body [49]. Liposomes that encapsulate microRNAs can provide solutions for major problems in small RNAs’ delivery, such as poor cellular uptake and facile enzymatic degradation.

In this study, we confirmed previously established effects of miR-203 constitutive overexpression and extended our analyses to include nanoliposome-mediated delivery. We assayed for effects on EMT status by analyzing migration and sphere formation as well as target transcript and protein expression in multiple cell lines. We find that both endogenous expression and exogenous delivery of a miR-203 synthetic mimic are sufficient to suppress cellular migration, independent of effects on EMT state, yet acting through distinct target mRNAs.

## 2. Results

### 2.1. Overexpression of miR-203 through Nanoliposomal Encapsulation

**Figure 1:**
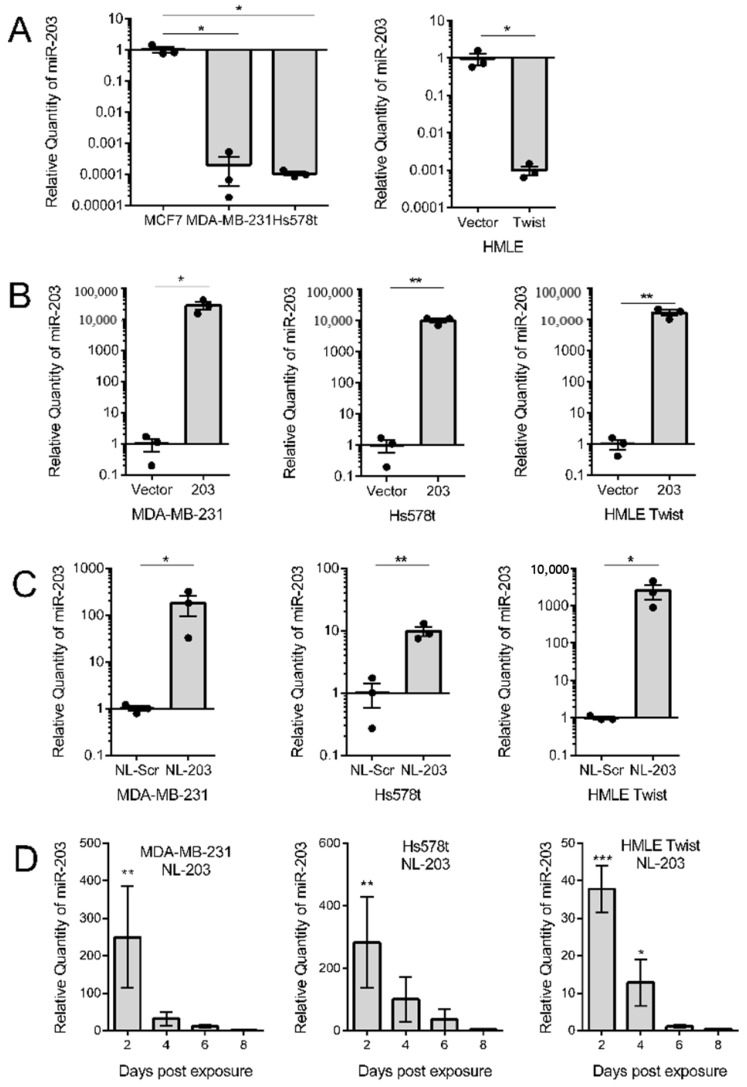
Relative expression of miR-203 performed by miRNA-specific quantitative RT-PCR. (**A**) miR-203 expression in breast cancer cell lines and in human mammary epithelial (HMLE) cells induced to undergo epithelial–mesenchymal transition (EMT) in response to Twist overexpression. (**B**) miR-203 expression in indicated cell lines transduced with a retrovirus vector to stably overexpress miR-203. (**C**) miR-203 expression in indicated cell lines exposed to microRNA mimic-loaded nanoliposomes at a concentration of 85 μM for 48 h. Data are presented as the mean and SEM of three independent replicates, two-tailed Student’s *t*-test. (**D**) miR-203 expression in indicated cell lines for the indicated number of days post-exposure to microRNA-203 mimic-loaded nanoliposomes. Expression is presented relative to control cells exposed to scramble-loaded nanoliposomes. Small nucleolar RNA U6 was used as a normalization control. Data are presented as the mean and SEM of three independent replicates, one-way analysis of variance (ANOVA) with Dunnet’s correction for multiple hypothesis testing. * *p* < 0.05, ** *p* < 0.01, *** *p* < 0.001.

It is well-established that epithelial–mesenchymal transition suppresses miR-203 expression. Likewise, the ability of restored miR-203 expression to suppress migration and sphere formation, two cellular phenotypes that correspond to cancer progression, has been established [31]. Yet, these studies have relied on constitutive overexpression of miR-203, and thus do not mimic what could be achievable in a clinical setting. To determine if more clinically relevant methodologies of microRNA-based therapy are also useful in facilitating the ability of miR-203 to impact cancer cells, we compared stable overexpression to nanoliposomal delivery. Our hypothesis being that miR-203 delivery suppresses migration and sphere formation through target gene downregulation irrespective of the delivery method. These experiments were conducted in two triple-negative breast cancer cell lines, MDA-MB-231 and Hs578t cells, and in our model of experimental EMT, HMLE-Twist cells (Figure 1A). Constitutive overexpression of miR-203 was accomplished using retrovirus transduction. Nanoliposomes were synthesized as previously described [48] and loaded with synthetic microRNA mimics of either a non-targeted scramble or miR-203. Through transmission electron microscopy and physicochemical characterization, we characterized that the liposomes encapsulated 96% of the RNA and their average particle size was 23.8 nm (Figure S1).

First, we determined whether the overexpression methods resulted in an increase in miR-203 expression. To do this, we conducted whole RNA extraction on replicates of all cell lines and performed miRNA-specific qRT-PCR to investigate the relative quantity of miR-203. As reference standards, HMLE and MCF7 cells were used as they have high endogenous expression of miR-203 (Figure 1A). miR-203 was found to be successfully overexpressed in all three cell lines (Figure 1B,C). To determine the duration of miR-203 transferred using nanoliposomes, we treated cells and then extracted RNA every two days post-treatment. The amount of miR-203 present in cells decreased over 50% between 2 and 4 days post-exposure and was indistinguishable from control cells by 6 days post-exposure (Figure 1D).

### 2.2. Overexpression and Delivery of miR-203 Suppress Migration

**Figure 2:**
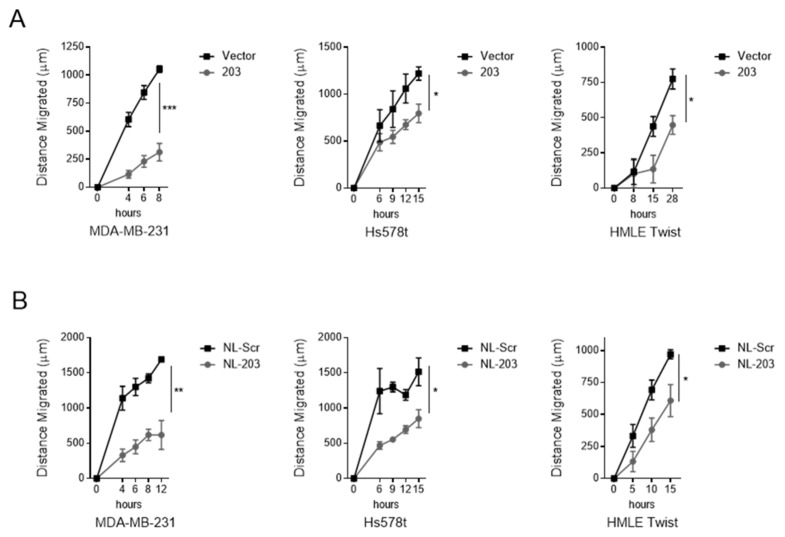
miR-203 inhibits migration. (**A**) Migration in indicated cell lines transduced with a retrovirus vector to stably overexpress miR-203. (**B**) Migration in indicated cell lines exposed to microRNA mimic-loaded nanoliposomes at a concentration of 85 μM for 48 h. Assay performed in quadruplicates. Represented as mean ± standard deviation of change in gap length from t = 0. *p*-value calculated for final measurement using Student’s two-tailed *t*-test. * *p* < 0.05, ** *p* < 0.01, *** *p* < 0.001.

The cell migration assay is a measure of cellular motility, which is enhanced in triple-negative breast cancer cells and following an EMT. We next evaluated whether miR-203 overexpression through either stable overexpression or exogenous delivery was sufficient to suppress the migratory capacity of target cells and whether the methodology of overexpression impacted the degree of suppression. To allow time for microRNA to alter cellular properties through target downregulation, cells were treated with nanoliposomes 48 h prior to plating for the migration assay. At specific time intervals, the distance between the two edges was measured at multiple positions. Stable overexpression of miR-203 was sufficient to suppress migration of MDA-MB-231 and Hs578t cells, consistent with our prior observation of the triple-negative breast cancer cell line SUM159 [31] (Figure 2A). Nanoliposomal delivery of miR-203 into HMLE Twist cells was also sufficient to suppress migration despite constitutive expression of the EMT-TF Twist (Figure 2B). In general, MDA-MB-231 cells were the most responsive to both methods of overexpressing miR-203. Our data suggest that restoring miR-203 expression levels inhibits migration in triple-negative breast cancer.

### 2.3. Delivery of miR-203 Suppresses Sphere Formation in HMLE Twist Cells

**Figure 3:**
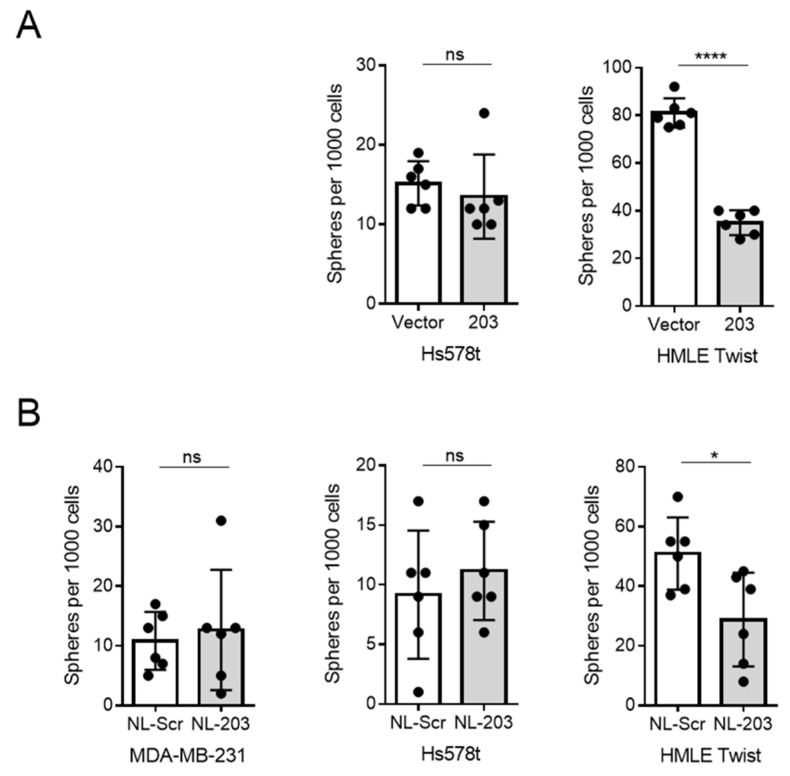
miR-203 is effective at inhibiting sphere formation in HMLE Twist cells, but not in triple negative breast cancer (TNBC) cell lines. (**A**) Sphere formation in indicated cell lines transduced with a retrovirus vector to stably overexpress miR-203. (**B**) Sphere formation in indicated cell lines exposed to microRNA mimic-loaded nanoliposomes at a concentration of 85 μM for 48 h prior to plating for sphere formation. Biological replicates, mean and standard deviation are represented. *p*-value calculated for final measurement using Student’s two-tailed *t*-test. ns = not significant; * *p* < 0.05, **** *p* < 0.0001.

Differentiated cells require anchoring to the extracellular matrix in order to proliferate. However, stem-like cells can divide and grow into spheroidal clusters of cells even under low-attachment conditions [50]. We have previously observed that constitutive overexpression of miR-203 was sufficient to suppress sphere formation, an indicator of stem-like properties, in SUM159 and HMLE Twist cells [31]. To test whether distinct methods of overexpressing miR-203 are sufficient to suppress stemness, we measured mammosphere formation. As expected, HMLE Twist cells were responsive to miR-203 constitutive expression; however, breast cancer cell lines were not responsive to miR-203 constitutive expression (Figure 3). HMLE Twist cells, nevertheless, showed a modest yet significant loss in stemness-associated sphere formation in response to nanoliposomal miR-203 (Figure 3). Thus, miR-203 delivery was sufficient to affect sphere formation, an in vitro measure of stemness, in an experimental model of EMT.

### 2.4. Overexpression and Delivery of miR-203 Alters Expression of Target mRNA

**Figure 4:**
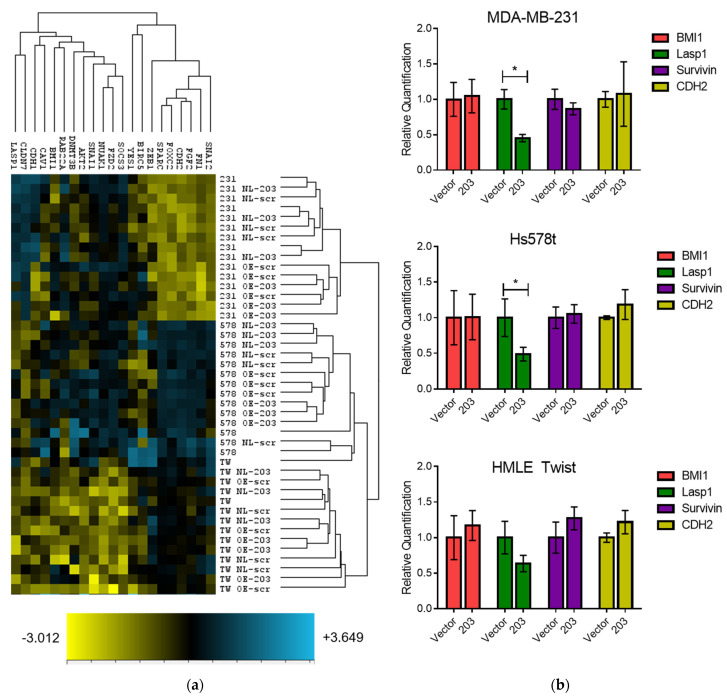

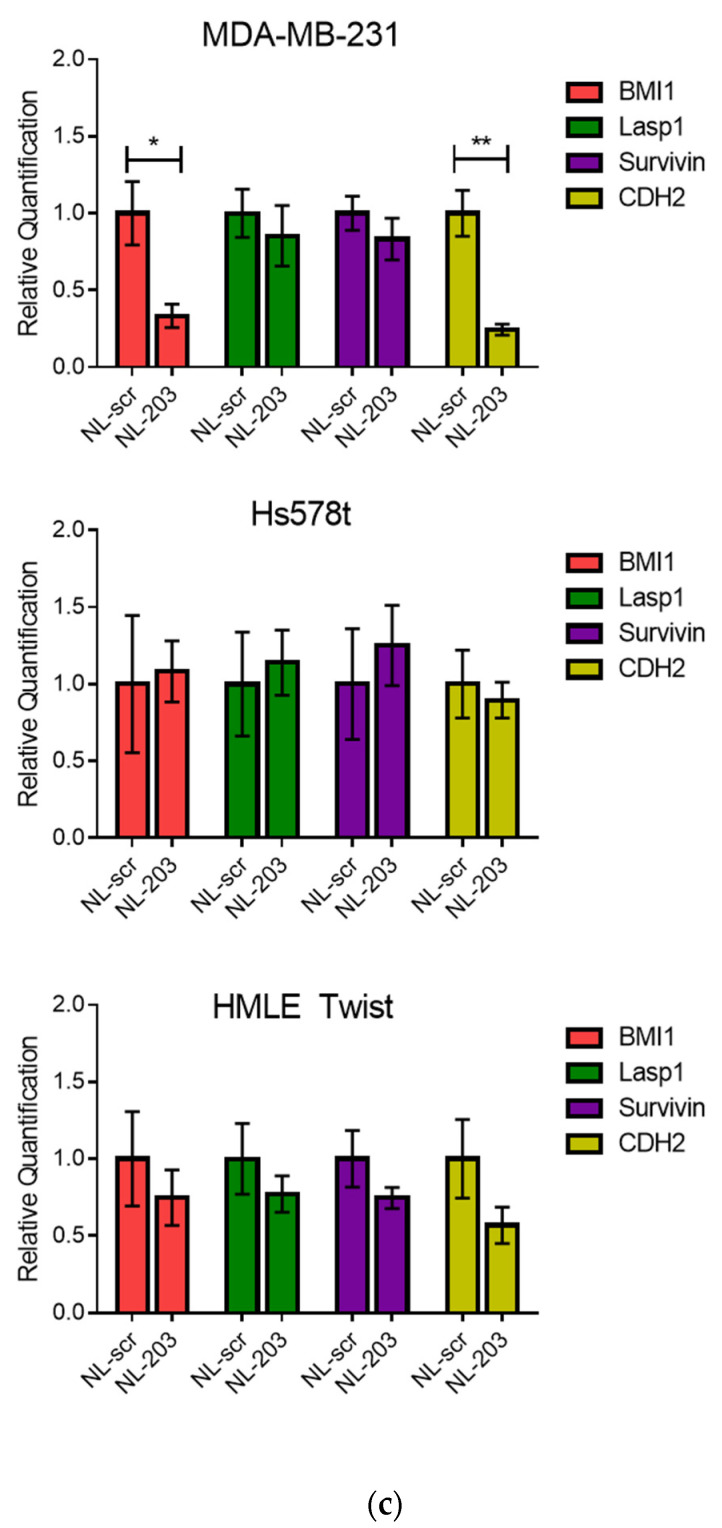
mRNA expression was measured by Nanostring PlexSet analysis and qRT-PCR. (**a**) Counts were normalized to three control genes and represented as z-scores within a hierarchical cluster. (**b**,**c**) Expression of indicated genes for cells with constitutive (**b**) or nanoliposomal-mediated (**c**) expression of miR-203 was determined using qRT-PCR. Mean and SEM of three biological replicates measured in technical quadruplicate are shown. Expression determined relative to vector or NL-scr control and normalized to GAPDH. *p*-value calculated using Student’s two-tailed *t*-test. * *p* < 0.05, ** *p* < 0.01.

In order to determine the effect of miR-203 overexpression on established mRNA targets and EMT markers, we measured the expression of a set of transcripts using direct enumeration through hybridization and fluorescent detection. Using the Nanostring PlexSet method, we measured the expression of 24 transcripts, including 3 housekeeping genes (GAPDH, HPRT1, and PKG1) combined with 15 established miR-203 targets (AKT2 [51], BIRC5 [37], BMI1 [33,34], DNMT3B [52], FGF2 [41], FZD2 [42], LASP1 [28,37], NUAK1 [28], RAB22A [53], SNAI2 [54,55], SOCS3 [56], SPARC [28], YES1 [57], and ZEB1 [29,35]) and 6 additional EMT-related genes (CAV1, CDH1, CDH2, CLDN7, FN1, FOXC2, and SNAI1) in cells with either constitutive or nanoliposomal-mediated miR-203 overexpression (in biological triplicate) (Figure 4a).

We noted changes in the expression of BMI1, LASP1, BIRC5 (survivin), and CDH2 in the Nanostring analysis. In order to quantitatively determine the effect of miR-203 overexpression on individual gene expression, we assessed expression of these genes using qRT-PCR. While most genes failed to show statistically significant differential expression, expression of LASP1 was consistently repressed by constitutive overexpression of miR-203 (Figure 4b), but not by nanoliposomal delivery (Figure 4c). However, nanoliposomal delivery of miR-203 resulted in suppression of BMI1 and CDH2, but only in MDA-MB-231 cells.

### 2.5. Overexpression of miR-203 Suppresses LASP1 and Survivin (BIRC5) Protein Expression, and Delivery of miR-203 Suppresses BMI1 Protein Expression

microRNAs can function through destabilization of their mRNA targets or suppression of protein translation. Therefore, changes in miR-203 target or EMT marker expression may or may not be evident at the transcript level. To determine if key targets were suppressed at the level of protein expression, we conducted western blot analysis.

**Figure 5:**
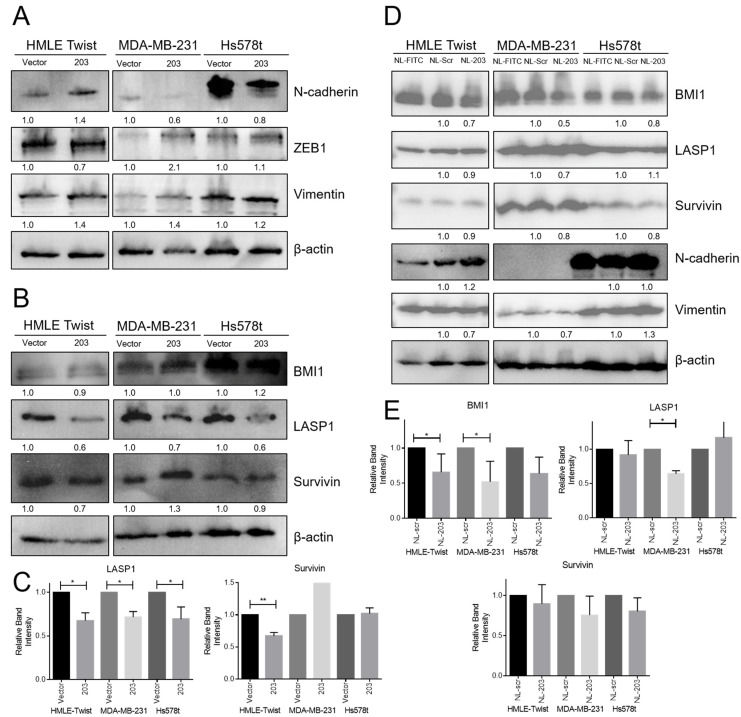
miR-203 induced changes in target proteins and EMT markers. Representative Western blots of indicated proteins from whole cell lysates from (**A**–**C**) indicated cells with stable miR-203 expression or (**D**,**E**) indicated cells exposed to microRNA mimic-, scramble control-, or FITC control-loaded nanoliposomes at a concentration of 85 μM for 48 h prior to protein extraction. (**C**,**E**) Band intensities from three biological replicates were quantified, normalized to β-actin, and then normalized to each cell line’s vector/scramble control. The average and standard deviation of three replicates are reported. *p*-value calculated using Student’s two-tailed *t*-test. * *p* < 0.05, ** *p* < 0.01.

As expected, constitutive miR-203 overexpression resulted in modest suppression of N-cadherin and vimentin but failed to induce upregulation of the epithelial marker, E-cadherin (Figure 5). This result is consistent with data from constitutive expression in SUM159 cells [31]. We next examined expression of select target proteins. Expression of LASP1, but not survivin or BMI1, was suppressed in all cell lines with constitutive miR-203 expression (Figure 5B,C) consistent with qRT-PCR data (Figure 4b). Remarkably, delivery of miR-203 by nanoliposome resulted in no change in expression of LASP1 or survivin, miR-203 targets, nor in N-cadherin or vimentin, EMT markers, but did result in a modest suppression of BMI1 which was also suppressed at the mRNA level--but only in MDA-MB-231 cells. This was surprising as miR-203 delivery by nanoliposome was sufficient to suppress migration in all cell lines tested and to suppress sphere formation in HMLE Twist cells. We thus conclude that miR-203 is capable of suppressing migration either through suppression of LASP1 and survivin or through suppression of BMI1.

### 2.6. Loss of MIR203 Copy Number in TNBC

**Figure 6:**
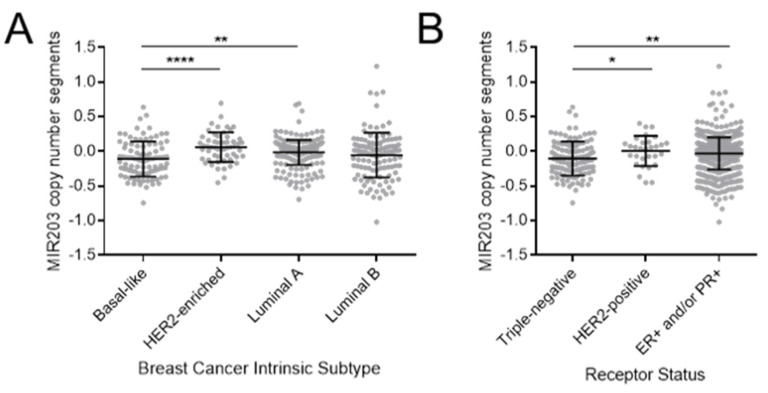
MIR203 copy number is reduced in basal-like and triple-negative breast cancers. Copy number data were obtained for patients with breast adenocarcinoma (BRCA) in the TCGA dataset using the UCSC Xena browser. Cases were stratified based on (**A**) intrinsic subtype according to the PAM50 method or (**B**) histological determination of estrogen receptor (ER), progesterone receptor (PR), or human epidermal growth factor receptor 2 (HER2) positivity. The mean and standard deviation are indicated by central bar and error bars. *p*-value calculated using Student’s two-tailed *t*-test. * *p* < 0.05, ** *p* < 0.01, **** *p* <0.0001.

A growing number of studies demonstrate genetic gain or loss of specific miRNAs in cancer [58]. To determine if miR-203 is gained or lost in specific breast cancer subtypes, we analyzed copy number data collected by The Cancer Genome Atlas database of breast carcinoma patients [59] and available using the UCSC Xena platform [60]. Consistent with the demonstrated lower expression in EMT-positive triple-negative breast cancer cell lines, patient data show a statistically significant decrease in copy number for the MIR203 gene in such cancers whether categorized based on intrinsic subtype, as in basal-like breast cancers, or based on histological analysis of receptor expression, as in triple-negative breast cancers (Figure 6).

In summary, expression miR-203 is demonstrated to impact cellular phenotypes associated with cancer disease progression including cellular migration and non-adherent cell growth. Moreover, the transient delivery of exogenous microRNA through nanoliposomal encapsulation was capable of mimicking some, though not all, of the effects of constitutive expression.

## 3. Discussion

miRNAs have many advantages as a therapeutic approach. Indeed, the pharmacological potential of miRNA mimics or inhibitors, demonstrated in animal disease models, suggests that miRNAs are viable targets or candidates for therapy [61,62]. Mature miRNA sequences are short and are usually completely conserved in multiple vertebrate species. These properties make miRNA relatively easy to target therapeutically and allow for the use of the same miRNA sequences in preclinical efficacy and safety studies as well as in clinical trials. In addition, miRNAs typically have many targets in cellular networks, which in turn can regulate entire pathways in disease states by targeting disease-associated mRNAs. A therapeutic strategy for restoring miRNA activity is to use synthetic double-stranded RNA that contains chemical modifications to improve stability and cellular uptake [63]. Major limitations associated with miRNA delivery are susceptibility to degradation by nucleases, rapid clearance from blood, immunotoxicity, and low tissue permeability [64,65]. miRNA mimics with liposome nanoparticles have been injected intravenously and intratumorally [66] in order to restore the function of various tumor-inhibiting miRNAs, such as miR-34a, in mouse cancer models [67]. However, unformulated miRNA mimics degrade rapidly in vivo. As such, an optimized liposome-encapsulated miRNA mimic has been shown to promote endocytosis and protect structures from degradation [68]. Neutral DOPC (1, 2-dioleoyl-tin-glycerol-3-phosphatidylcholine) liposomes were able to deliver miRNA-506 mimics or miRNA-520 in mouse models of ovarian cancer, resulting in significant tumor suppression [69]. Moreover, the recently demonstrated safety of the lipid nanoparticles used to deliver mRNA for vaccination highlights the potential for RNA-based therapeutics [70]. Taken together, these studies suggest that the programmability of small non-coding RNA nanostructures offers great promise for further exploration of miRNA-based therapies.

Several studies have reported a role of miR-203 as a tumor or metastasis suppressor in a variety of cancers including lung cancer [71] and breast cancer [72]. In recent years, a large number of studies have shown that promoter hypermethylation leads to down-regulation of miR-203 in several cancer types. Diao et al. reported that miR-203 hypermethylation was a common event in endometrial cancer and was closely related to microsatellite instability [26]. In addition, Huang et al. found that miR-203 was often downregulated by promoter hypermethylation in rhabdomyosarcoma cell lines and clinical biopsies, and the re-expression of miR-203 through DNA demethylation drugs inhibited the migration and proliferation of cancer cells and promoted final muscle-derived differentiation [73].

We tested the ability of re-introduced miR-203 to affect cellular properties associated with EMT and stemness and to affect gene expression. Our data indicate a consistent effect on the migration of target cells despite little effect on the EMT status. Nevertheless, not all cell lines respond identically to miR-203 restoration. In particular, miR-203 delivery via nanoliposome was sufficient to reduce sphere formation, a measure of stemness, in HMLE Twist cells, but not either breast cancer cell line. Cancer cell lines often display redundant pathway activation, which may contribute to the failure of these cells to respond. Nevertheless, SUM159 cells, another TNBC cell line, do exhibit diminished stemness features in response to stable miR-203 expression [31]. Nanoliposomal delivery of miR-203 significantly impacts cellular migration, yet does not suppress LASP1, which is consistently suppressed by stable expression of miR-203. The most consistent molecular change associated with nanoliposomal miR-203 is suppression of BMI1 protein, in MDA-MB-231 and Hs578t cells, and BMI1 mRNA, in MDA-MB-231 cells. BMI1, a member of the PRC2 complex that regulates chromatin modification, has been linked to EMT, migration, and metastasis [74,75]. Defining the relationship between cell type and molecular response to the recovery of miR-203 will help reveal the specific mechanisms of miR-203 action. This work supports further research on the use of miR-203 or small molecules sufficient to relieve miR-203 repression as a therapeutic for triple-negative breast cancer.

## 4. Materials and Methods

### 4.1. Cell Culture

Human triple-negative breast cancer cells MCF7, MDA-MB-231, and Hs578t (ATCC) were cultured in Dulbecco’s modified Eagle medium (DMEM) (Corning Inc., Kennebuck, ME, USA) supplemented with 10% fetal bovine serum (FBS) (Equitech-Bio Inc., Kerrville, TX, USA), 1% penicillin/streptomycin (Lonza, Basel, Switzerland). Immortalized human mammary epithelial cells (HMLE and HMLE Twist) were cultured in DMEM/F12 medium (GE Healthcare Life Sciences, Chicago, IL, USA) supplemented with 5 μg/mL insulin (MilliporeSigma, St. Louis, MO, USA), 0.01 μg/mL hEGF (MilliporeSigma), 0.5 μg/mL hydrocortisone (Acros Organics, Fair Lawn, NJ, USA), and 1% penicillin/streptomycin, mixed with MEGM supplemented with BPE (Lonza, Basel, Switzerland). All cell lines were cultured at 37 °C in 5% CO_2_, and the media was replaced every three days.

### 4.2. Viral Transduction

MIR203 was cloned into the pBabe expression vector using primers F: 5′-agtcggatccctcctctctccgcagctc-3′ and R: 5′-agtcgaattcgcacccctgactgtgactct-3′. Bacteria containing the vectors were grown in autoclaved Luria–Bertani (LB) broth with ampicillin for selection. Plasmid purification was done using the GeneJET Plasmid Midi Prep kit (ThermoFisher, Waltham, MA, USA) according to the manual instruction. For virus production, 1.5 × 10^6^ 293T cells were plated onto 15 cm culture dishes in 20 mL culture media. Transfection took place after 12–20 h or when cells were about 50–60% confluent. Transfection mix included 200 mL media, 1.8 mg Δ8.2, 0.2 mg pCMV-VSVG, and 2.0 mg of control or miR-203 expressing vectors and 15 mL Fugene HD (Promega, Madison, WI, USA). Transfection mix was incubated at room temperature for 15 min, but no longer than 45 min before being dripped directly onto 293T cells and incubated overnight at 37 °C. The next day, 293T cells were supplied with 15 mL fresh media (4:1 ratio of target cell media to 293T cells media). Target cells, HMLE Twist, MDA-MB-231, and Hs578t cells were seeded or transfection at 30–50% confluency. Next, we harvested the supernatants 48 and 72 h after transfection and passed the supernatants through a 0.45-micron filter. Target cells were transduced with viral supernatants (3 mL per 6 cm plate) and protamine sulfate at a final concentration of 4 mg/mL. After 24 to 48 h of incubation with virus, infected cells were selected with 2 μg/mL puromycin or through sorting for GFP-positivity using the FACS Melody (BD Biosciences, Franklin Lakes, NJ, USA). Transduced cell populations were expanded in culture for the minimal time period to obtain a sufficient number of cells to set up replicate experiments.

### 4.3. Nanoliposome Synthesis

Didodecyldimethylammonium bromide (DDAB; Millipore Sigma, St. Louis, MO, USA), ovine cholesterol (Avanti Polar Lipids, Alabaster, AL, USA), and tocopherol PEG 1000 succinate (TPGS; Millipore Sigma, St. Louis, MO, USA) were each suspended in 10 mL of 100% ethanol in different vials at molar ratios of 12, 7, and 1 M, respectively (i.e., 40.65 mg DDAB, 22.55 mg cholesterol, and 12.60 mg TPGS). In a new vial, 1 mL of each suspended reagent was mixed together and then 431 μL of this pre-liposome solution was aliquoted into clean vials for incubation with RNA samples. Next, the fluorescently tagged siRNA (Santa Cruz Biotechnology, Dallas, TX, USA), synthetic (hsa-mir-203) microRNA, negative control RNA (MISSION^®^, Millipore Sigma, St. Louis, MO, USA), or FITC-labeled scramble RNA was resuspended in RNase free water as described by the manufacturer. Each RNA sample was then incubated at room temperature (22°) for 30 min in an aliquot of pre-liposome solution. To a separate vial containing 4.569 mL of PBS, being magnetically stirred at 1400 RPM, the RNA/pre-liposome working solution was pulled into a glass syringe and then rapidly injected. After 5 min of vigorous stirring, the final liposome solution underwent dialysis in a Float-A-Lyzer (8–10 kDa size; Spectrum Laboratories, Houston, TX, USA) overnight and then stored in a clean scintillation vial at 4° until use. The final concentration of each solution is 0.5 mM liposome with 20 nm of RNA sample.

When the cells reached 80–90% confluence, the media was replenished with fresh culture media and loaded nanoliposomes in PBS were directly added in culture media to the cells at a final concentration of 85 μM and cultured in the incubator for 48 h.

### 4.4. RNA Isolation and Quantitative Reverse-Transcription PCR

Total RNA in cells was isolated using Trizol^®^ Reagent (Thermo Scientific, Waltham, MA, USA) according to the manufacturer’s protocol. The amount and purity of isolation RNA were analyzed by the Nanodrop spectrophotometer. Complementary DNA was synthesized using Reverse Transcriptase. qPCR was performed as quadruplicate using Taqman Master mix and miRNA-specific primers (Applied Biosystems, Foster City, CA, USA; Thermo Scientific). Thermal cycle conditions used for all qPCR reactions were as follows: 10 min at 95 °C, followed by 40 cycles consisting between denaturation for 15 s at 95 °C and annealing for 60 s at 60 °C. PCR reactions were concluded with incubation for 10 min at 72 °C to compete the extension of all synthesized products QuantStudio 5 (Applied Biosystems). The expression of miRNAs was normalized against that of U6, and relative quantification values were calculated using the 2-ΔΔCt method.

### 4.5. Nanostring Analysis

Total RNA isolated as above was subjected to analysis using a PlexSet probe set. A total of 450 ng of RNA in TE buffer was hybridized to probes for AKT2, BIRC5, BMI1, CAV1, CDH1, CDH2, CLDN7, DNMT3B, FGF2, FN1, FOXC2, FZD2, LASP1, NUAK1, RAB22A, SNAI1, SNAI2, SOCS3, SPARC, YES1, ZEB1, GAPDH, HPRT1, and PKG1 provided by Nanostring (Nanostring Technologies, Seattle WA, USA). Hybridization was conducted on 96 RNA samples, using a multiplexing arrangement, for 16 h at 65° according to the manufacturer’s protocol. Raw counts were normalized to the geometric mean of the count values for the three endogenous control genes, GAPDH, HPRT1, and PKG1. Expression across samples was computed as a z-score using the formula Z = (x − μ)/σ where x = the observed value, μ = the mean across all samples, and σ = the standard deviation across all samples. Hierarchical clustering is based on Euclidean distance (nSolver 4.0).

### 4.6. Immunoblotting

Cells were lysed in ice-cold radio-immunoprecipitation (RIPA) buffer containing protease inhibitor and phosphatase inhibitor cocktail (Alfa Aesar, Stoughton, MA, USA). Protein was quantified using the Bradford Assay (BioRad, Hercules, CA, USA). Proteins (20–30 μg) were resolved in SDS-polyacrylamide gels (10%) and transferred to PVDF membranes using a Tris-glycine buffer system. Membranes were blocked with 5% non-fat dry milk in 0.1% Tween20 in TBS (TBST) for 1 h at room temperature followed by probing with primary antibodies and gently rocking overnight at 4 °C. Antibodies used for immunoblotting were Mouse anti-E-cadherin (Cell Signaling, Danvers, MA, USA), Rabbit anti-N-cadherin (Cell Signaling), Rabbit anti-vimentin (Protein Technologies, Tucson, AZ, USA), Rabbit anti-survivin (Cell Signaling), Rabbit anti-Lasp1 (Cell Signaling), Rabbit anti-BMI1 (Cell Signaling), and Mouse anti-β-actin (BD Biosciences, San Jose, CA, USA). The next day, the blots were washed with TBST three times, 5 min each time. They were incubated with secondary antibodies (1:2000) at room temperature for 1 h. Chemiluminescent signals were detected with ECL™ prime (Thermo Fisher Scientific, Waltham, MA, USA) using the Biorad ChemiDoc system. If necessary, blots were stripped with ECL Stripping Buffer (Li-Cor, Lincoln, NB, USA) following the manufacturer’s protocol. Bands were quantified using ImageJ.

### 4.7. Cell Migration Assay

To perform the cell migration assay, we first drew two parallel horizonal lines upon the bottom of a 24-well plate before seeding cells into it. Then, cells were added to new media up to 0.5 mL/well in a 24-well plate, at a final seeding density as 5 × 10^4^ cells per well. The next day, the media was replenished gently, and the well was scratched with a straight line on the cell monolayer across the center of each well with a 200 μL micropipette tip. At regular intervals, we imaged the gaps of each well. Cell migration rates were determined by measuring the distance between the cell frontiers after a specified culture hour. At a given point in time, the distance between the two edges at multiple points was quantified using GraphPad Prism 6.0. The change in gap length is plotted over time.

### 4.8. Mammosphere Assay

Cells were harvested according to standard protocol and resuspended in serum-free mammary epithelial growth medium (MEGM) supplemented with 20 ng/mL bFGF, 10 ng/mL EGF, 4 ug/mL heparin, and 1% Methylcellulose. Then, 1000 cells per well were plated in a 96-well ultra-low attachment plate (Corning, Corning, NY, USA) in 100 μL mammosphere media. Every third day, 100 μL of fresh mammosphere media was added into each well. Spheres formed around 10–14 days. Wells were imaged using 10× magnification on a computer-assisted phase contrast microscope (Nikon, Tokyo, Japan). Rounded spheroids greater than 50 μm were considered as mammospheres.

### 4.9. Patient Analysis

To evaluate the copy number of MIR203 in breast cancer, we acquired the genomic data for 1247 breast tumor samples in TCGA by downloading the log2(tumor/normal) values for copy number, calculated after removal of germline copy number variation. We distinguished the tumors into triple-negative or others using the clinical annotations provided by the TCGA. We compared the copy number levels between groupings within Graphpad Prism 6.0 using a one-way ANOVA accompanied by pair-wise comparisons modified by Dunnet’s correction for multiple hypothesis testing.

### 4.10. Statistical Analysis

Data were analyzed using Prism 6 (GraphPad) software. Statistical significance is indicated as * *p* < 0.05, ** *p* < 0.01, *** *p* < 0.001, or ns (not significant), using Student’s unpaired two-tailed *t*-test. Data were normalized in some cases. Either single values per cell line or mean ± standard division are plotted throughout the manuscript. All experiments were performed at least three independent times.

## Data Availability

Not applicable.

## Supplementary Materials

The following are available online at https://www.mdpi.com/article/10.3390/ncrna7030045/s1, Figure S1: Physicochemical characterization of the nanoliposomes used in this study. (a) transmission electron micrograph (TEM) of nanoliposome population. Particle agglomeration is moderate because liposomes easily disperse when in aqueous suspension. (b) quantitative data collected from TEM, dynamic light scattering (DLS), and fluorescence spectroscopy. Data was collected in triplicate over four different experiments (n = 12). For particle size analysis, only individual lipo-somes were measured.

## References

[1] American Cancer Society (2021). Breast Cancer Facts & Figures.

[2] Pareja F., Geyer F.C., Marchiò C., Burke K.A., Weigelt B., Reis-Filho J.S. (2016). Triple-negative breast cancer: The importance of molecular and histologic subtyping, and recognition of low-grade variants. NPJ Breast Cancer.

[3] Lu W., Kang Y. (2019). Epithelial-Mesenchymal Plasticity in Cancer Progression and Metastasis. Dev. Cell.

[4] Tran H.D., Luitel K., Kim M., Zhang K., Longmore G.D., Tran D.D. (2014). Transient SNAIL1 expression is necessary for metastatic competence in breast cancer. Cancer Res..

[5] Xu Y., Lee D.K., Feng Z., Xu Y., Bu W., Li Y., Liao L., Xu J. (2017). Breast tumor cell-specific knockout of Twist1 inhibits cancer cell plasticity, dissemination, and lung metastasis in mice. Proc. Natl. Acad. Sci. USA.

[6] Revenco T., Nicodeme A., Pastushenko I., Sznurkowska M.K., Latil M., Sotiropoulou P.A., Dubois C., Moers V., Lemaire S., de Maertelaer V. (2019). Context Dependency of Epithelial-to-Mesenchymal Transition for Metastasis. Cell Rep..

[7] Friedl P., Mayor R. (2017). Tuning collective cell migration by cell-cell junction regulation. Cold Spring Harb. Perspect. Biol..

[8] Nieto M.A., Huang R.Y., Jackson R.A., Thiery J.P. (2016). Emt: 2016. Cell.

[9] Herranz N., Pasini D., Diaz V.M., Franci C., Gutierrez A., Dave N., Escriva M., Hernandez-Munoz I., Di Croce L., Helin K. (2008). Polycomb complex 2 is required for E-cadherin repression by the Snail1 transcription factor. Mol. Cell Biol..

[10] Lamouille S., Xu J., Derynck R. (2014). Molecular mechanisms of epithelial-mesenchymal transition. Nat. Rev. Mol. Cell Biol..

[11] Werden S.J., Sphyris N., Sarkar T.R., Paranjape A.N., LaBaff A.M., Taube J.H., Hollier B.G., Ramirez-Pena E.Q., Soundararajan R., den Hollander P. (2016). Phosphorylation of serine 367 of FOXC2 by p38 regulates ZEB1 and breast cancer metastasis, without impacting primary tumor growth. Oncogene.

[12] Comijn J., Berx G., Vermassen P., Verschueren K., van Grunsven L., Bruyneel E., Mareel M., Huylebroeck D., van Roy F. (2001). The two-handed E box binding zinc finger protein SIP1 downregulates E-cadherin and induces invasion. Mol. Cell.

[13] Krebs A.M., Mitschke J., Lasierra Losada M., Schmalhofer O., Boerries M., Busch H., Boettcher M., Mougiakakos D., Reichardt W., Bronsert P. (2017). The EMT-activator Zeb1 is a key factor for cell plasticity and promotes metastasis in pancreatic cancer. Nat. Cell Biol..

[14] Stemmler M.P., Eccles R.L., Brabletz S., Brabletz T. (2019). Non-redundant functions of EMT transcription factors. Nat. Cell Biol..

[15] Bartel D.P. (2009). MicroRNAs: Target recognition and regulatory functions. Cell.

[16] Lee Y.S., Dutta A. (2009). MicroRNAs in cancer. Annu. Rev. Pathol..

[17] Filipowicz W., Bhattacharyya S.N., Sonenberg N. (2008). Mechanisms of post-transcriptional regulation by microRNAs: Are the answers in sight?. Nat. Rev. Genet..

[18] Di Leva G., Garofalo M., Croce C.M. (2014). MicroRNAs in cancer. Annu. Rev. Pathol..

[19] Bracken C.P., Gregory P.A., Kolesnikoff N., Bert A.G., Wang J., Shannon M.F., Goodall G.J. (2008). A double-negative feedback loop between ZEB1-SIP1 and the microRNA-200 family regulates epithelial-mesenchymal transition. Cancer Res..

[20] Title A.C., Hong S.-J., Pires N.D., Hasenöhrl L., Godbersen S., Stokar-Regenscheit N., Bartel D.P., Stoffel M. (2018). Genetic dissection of the miR-200-Zeb1 axis reveals its importance in tumor differentiation and invasion. Nat. Commun..

[21] Zaravinos A. (2015). The Regulatory Role of MicroRNAs in EMT and Cancer. J. Oncol..

[22] Brabletz S., Brabletz T. (2010). The ZEB/miR-200 feedback loop--a motor of cellular plasticity in development and cancer?. EMBO Rep..

[23] Burk U., Schubert J., Wellner U., Schmalhofer O., Vincan E., Spaderna S., Brabletz T. (2008). A reciprocal repression between ZEB1 and members of the miR-200 family promotes EMT and invasion in cancer cells. EMBO Rep..

[24] Chim C.S., Wong K.Y., Leung C.Y., Chung L.P., Hui P.K., Chan S.Y., Yu L. (2011). Epigenetic inactivation of the hsa-miR-203 in haematological malignancies. J. Cell Mol. Med..

[25] Liao H., Bai Y., Qiu S., Zheng L., Huang L., Liu T., Wang X., Liu Y., Xu N., Yan X. (2015). MiR-203 downregulation is responsible for chemoresistance in human glioblastoma by promoting epithelial-mesenchymal transition via SNAI2. Oncotarget.

[26] Diao Y., Guo X., Jiang L., Wang G., Zhang C., Wan J., Jin Y., Wu Z. (2014). miR-203, a tumor suppressor frequently down-regulated by promoter hypermethylation in rhabdomyosarcoma. J. Biol. Chem..

[27] Saini S., Majid S., Yamamura S., Tabatabai L., Suh S.O., Shahryari V., Chen Y., Deng G., Tanaka Y., Dahiya R. (2011). Regulatory Role of mir-203 in Prostate Cancer Progression and Metastasis. Clin. Cancer Res..

[28] Benaich N., Woodhouse S., Goldie S.J., Mishra A., Quist S.R., Watt F.M. (2014). Rewiring of an Epithelial Differentiation Factor, miR-203, to Inhibit Human Squamous Cell Carcinoma Metastasis. Cell Rep..

[29] Meidhof S., Brabletz S., Lehmann W., Preca B.-T., Mock K., Ruh M., Schüler J., Berthold M., Weber A., Burk U. (2015). ZEB1-associated drug resistance in cancer cells is reversed by the class I HDAC inhibitor mocetinostat. EMBO Mol. Med..

[30] Bueno M.J., Perez de Castro I., Gomez de Cedron M., Santos J., Calin G.A., Cigudosa J.C., Croce C.M., Fernandez-Piqueras J., Malumbres M. (2008). Genetic and epigenetic silencing of microRNA-203 enhances ABL1 and BCR-ABL1 oncogene expression. Cancer Cell.

[31] Taube J.H., Malouf G.G., Lu E., Sphyris N., Vijay V., Ramachandran P.P., Ueno K.R., Gaur S., Nicoloso M.S., Rossi S. (2013). Epigenetic silencing of microRNA-203 is required for EMT and cancer stem cell properties. Sci. Rep..

[32] Ge X., Li G.Y., Jiang L., Jia L., Zhang Z., Li X., Wang R., Zhou M., Zhou Y., Zeng Z. (2019). Long noncoding RNA CAR10 promotes lung adenocarcinoma metastasis via miR-203/30/SNAI axis. Oncogene.

[33] Patel N., Garikapati K.R., Makani V.K.K., Nair A.D., Vangara N., Bhadra U., Bhadra M.P. (2018). Regulating BMI1 expression via miRNAs promote Mesenchymal to Epithelial Transition (MET) and sensitizes breast cancer cell to chemotherapeutic drug. PLoS ONE.

[34] Chen T.F., Xu C., Chen J., Ding C., Xu Z.L., Li C., Zhao J. (2015). MicroRNA-203 inhibits cellular proliferation and invasion by targeting Bmi1 in non-small cell lung cancer. Oncol. Lett..

[35] Wellner U., Schubert J., Burk U.C., Schmalhofer O., Zhu F., Sonntag A., Waldvogel B., Vannier C., Darling D., zur Hausen A. (2009). The EMT-activator ZEB1 promotes tumorigenicity by repressing stemness-inhibiting microRNAs. Nat. Cell Biol..

[36] Ungvári I., Hadadi E., Virág V., Bikov A., Nagy A., Semsei A.F., Gálffy G., Tamási L., Horváth I., Szalai C. (2012). Implication of BIRC5 in asthma pathogenesis. Int. Immunol..

[37] Wang C., Zheng X., Shen C., Shi Y. (2012). MicroRNA-203 suppresses cell proliferation and migration by targeting BIRC5 and LASP1 in human triple-negative breast cancer cells. J. Exp. Clin. Cancer Res..

[38] Hailer A., Grunewald T.G.P., Orth M., Reiss C., Kneitz B., Spahn M., Butt E. (2014). Loss of tumor suppressor mir-203 mediates overexpression of LIM and SH3 Protein 1 (LASP1) in high-risk prostate cancer thereby increasing cell proliferation and migration. Oncotarget.

[39] Zhou Y., Wan G., Spizzo R., Ivan C., Mathur R., Hu X., Ye X., Lu J., Fan F., Xia L. (2014). miR-203 induces oxaliplatin resistance in colorectal cancer cells by negatively regulating ATM kinase. Mol. Oncol..

[40] Shen J., Zhang J., Xiao M., Yang J., Zhang N. (2018). miR-203 Suppresses Bladder Cancer Cell Growth and Targets Twist1. Oncol. Res..

[41] He S., Zhang G., Dong H., Ma M., Sun Q. (2016). miR-203 facilitates tumor growth and metastasis by targeting fibroblast growth factor 2 in breast cancer. OncoTargets Ther..

[42] Mine M., Yamaguchi K., Sugiura T., Chigita S., Yoshihama N., Yoshihama R., Hiyake N., Kobayashi Y., Mori Y. (2015). miR-203 Inhibits Frizzled-2 Expression via CD82/KAI1 Expression in Human Lung Carcinoma Cells. PLoS ONE.

[43] Shi J., Kantoff P.W., Wooster R., Farokhzad O.C. (2017). Cancer nanomedicine: Progress, challenges and opportunities. Nat. Rev. Cancer.

[44] Wang J., Wu X., Shen P., Wang J., Shen Y., Shen Y., Webster T.J., Deng J. (2020). Applications of Inorganic Nanomaterials in Photothermal Therapy Based on Combinational Cancer Treatment. Int. J. Nanomed..

[45] Malam Y., Loizidou M., Seifalian A.M. (2009). Liposomes and nanoparticles: Nanosized vehicles for drug delivery in cancer. Trends Pharmacol. Sci..

[46] Etheridge M.L., Campbell S.A., Erdman A.G., Haynes C.L., Wolf S.M., McCullough J. (2013). The big picture on nanomedicine: The state of investigational and approved nanomedicine products. Nanomedicine.

[47] Prabhakar U., Maeda H., Jain R.K., Sevick-Muraca E.M., Zamboni W., Farokhzad O.C., Barry S.T., Gabizon A., Grodzinski P., Blakey D.C. (2013). Challenges and key considerations of the enhanced permeability and retention effect for nanomedicine drug delivery in oncology. Cancer Res..

[48] Lujan H., Griffin W.C., Taube J.H., Sayes C.M. (2019). Synthesis and characterization of nanometer-sized liposomes for encapsulation and microRNA transfer to breast cancer cells. Int. J. Nanomed..

[49] Chang H.I., Yeh M.K. (2012). Clinical development of liposome-based drugs: Formulation, characterization, and therapeutic efficacy. Int. J. Nanomed..

[50] Dontu G., Abdallah W.M., Foley J.M., Jackson K.W., Clarke M.F., Kawamura M.J., Wicha M.S. (2003). In vitro propagation and transcriptional profiling of human mammary stem/progenitor cells. Genes Dev..

[51] Li J., Chen Y., Zhao J., Kong F., Zhang Y. (2011). miR-203 reverses chemoresistance in p53-mutated colon cancer cells through downregulation of Akt2 expression. Cancer Lett..

[52] To K.K., Leung W.W., Ng S.S. (2016). A novel miR-203-DNMT3b-ABCG2 regulatory pathway predisposing colorectal cancer development. Mol. Carcinog..

[53] Yang D.W., Liu G.P., Wang K.Z. (2015). miR-203 acts as a tumor suppressor gene in osteosarcoma by regulating RAB22A. PLoS ONE.

[54] Tian X., Tao F., Zhang B., Dong J.-T., Zhang Z. (2018). The miR-203/SNAI2 axis regulates prostate tumor growth, migration, angiogenesis and stemness potentially by modulating GSK-3β/β-CATENIN signal pathway. IUBMB Life.

[55] Zhang Z., Zhang B., Li W., Fu L., Fu L., Zhu Z., Dong J.T. (2011). Epigenetic Silencing of miR-203 Upregulates SNAI2 and Contributes to the Invasiveness of Malignant Breast Cancer Cells. Genes Cancer.

[56] Ru P., Steele R., Hsueh E.C., Ray R.B. (2011). Anti-mir-203 upregulates socs3 expression in breast cancer cells and enhances cisplatin chemosensitivity. Genes Cancer.

[57] Lee S.A., Kim J.S., Park S.Y., Kim H.J., Yu S.K., Kim C.S., Chun H.S., Kim J., Park J.T., Go D. (2015). miR-203 downregulates Yes-1 and suppresses oncogenic activity in human oral cancer cells. J. Biosci. Bioeng..

[58] Peng Y., Croce C.M. (2016). The role of MicroRNAs in human cancer. Signal Transduct. Target. Ther..

[59] Koboldt D.C.F.R., Fulton R., McLellan M., Schmidt H., Kalicki-Veizer J., McMichael J., Fulton L., Dooling D., Ding L., Mardis E. (2012). Comprehensive molecular portraits of human breast tumours. Nature.

[60] Goldman M.J., Craft B., Hastie M., Repecka K., McDade F., Kamath A., Banerjee A., Luo Y.H., Rogers D., Brooks A.N. (2020). Visualizing and interpreting cancer genomics data via the Xena platform. Nat. Biotechnol..

[61] Stenvang J., Petri A., Lindow M., Obad S., Kauppinen S. (2012). Inhibition of microRNA function by antimiR oligonucleotides. Silence.

[62] Thorsen S.B., Obad S., Jensen N.F., Stenvang J., Kauppinen S. (2012). The therapeutic potential of microRNAs in cancer. Cancer J..

[63] Garzon R., Marcucci G., Croce C.M. (2010). Targeting microRNAs in cancer: Rationale, strategies and challenges. Nat. Rev. Drug Discov..

[64] Grimm D., Streetz K.L., Jopling C.L., Storm T.A., Pandey K., Davis C.R., Marion P., Salazar F., Kay M.A. (2006). Fatality in mice due to oversaturation of cellular microRNA/short hairpin RNA pathways. Nature.

[65] Wang H., Jiang Y., Peng H., Chen Y., Zhu P., Huang Y. (2015). Recent progress in microRNA delivery for cancer therapy by non-viral synthetic vectors. Adv. Drug Deliv. Rev..

[66] Pramanik D., Campbell N.R., Karikari C., Chivukula R., Kent O.A., Mendell J.T., Maitra A. (2011). Restitution of tumor suppressor microRNAs using a systemic nanovector inhibits pancreatic cancer growth in mice. Mol. Cancer Ther..

[67] He L., He X., Lim L.P., de Stanchina E., Xuan Z., Liang Y., Xue W., Zender L., Magnus J., Ridzon D. (2007). A microRNA component of the p53 tumour suppressor network. Nature.

[68] Wiggins J.F., Ruffino L., Kelnar K., Omotola M., Patrawala L., Brown D., Bader A.G. (2010). Development of a lung cancer therapeutic based on the tumor suppressor microRNA-34. Cancer Res..

[69] Nishimura M., Jung E.J., Shah M.Y., Lu C., Spizzo R., Shimizu M., Han H.D., Ivan C., Rossi S., Zhang X. (2013). Therapeutic synergy between microRNA and siRNA in ovarian cancer treatment. Cancer Discov..

[70] Polack F.P., Thomas S.J., Kitchin N., Absalon J., Gurtman A., Lockhart S., Perez J.L., Marc G.P., Moreira E.D., Zerbini C. (2020). Safety and Efficacy of the BNT162b2 mRNA Covid-19 Vaccine. N. Engl. J. Med..

[71] Wang N., Liang H., Zhou Y., Wang C., Zhang S., Pan Y., Wang Y., Yan X., Zhang J., Zhang C.Y. (2014). miR-203 suppresses the proliferation and migration and promotes the apoptosis of lung cancer cells by targeting SRC. PLoS ONE.

[72] Orom U.A., Lim M.K., Savage J.E., Jin L., Saleh A.D., Lisanti M.P., Simone N.L. (2012). MicroRNA-203 regulates caveolin-1 in breast tissue during caloric restriction. Cell Cycle.

[73] Huang Y.W., Kuo C.T., Chen J.H., Goodfellow P.J., Huang T.H., Rader J.S., Uyar D.S. (2014). Hypermethylation of miR-203 in endometrial carcinomas. Gynecol. Oncol..

[74] Yang M.H., Hsu D.S.S., Wang H.W., Wang H.J., Lan H.Y., Yang W.H., Huang C.H., Kao S.Y., Tzeng C.H., Tai S.K. (2010). Bmi1 is essential in Twist1-induced epithelial-mesenchymal transition. Nat. Cell Biol..

[75] Paranjape A.N., Balaji S.A., Mandal T., Krushik E.V., Nagaraj P., Mukherjee G., Rangarajan A. (2014). Bmi1 regulates self-renewal and epithelial to mesenchymal transition in breast cancer cells through Nanog. BMC Cancer.

