# Supplementary material for: Nanoliposomal Delivery of MicroRNA-203 Suppresses Migration of Triple-Negative Breast Cancer through Distinct Target Suppression
Source: Noncoding RNA. 2021 Jul 27;7(3):45. doi: 10.3390/ncrna7030045 (PMC8395754; doi:10.3390/ncrna7030045)
Supplement: Supplementary file 1 [file ncrna-07-00045-s001.zip › ncrna-1236427-supplementary.pdf]

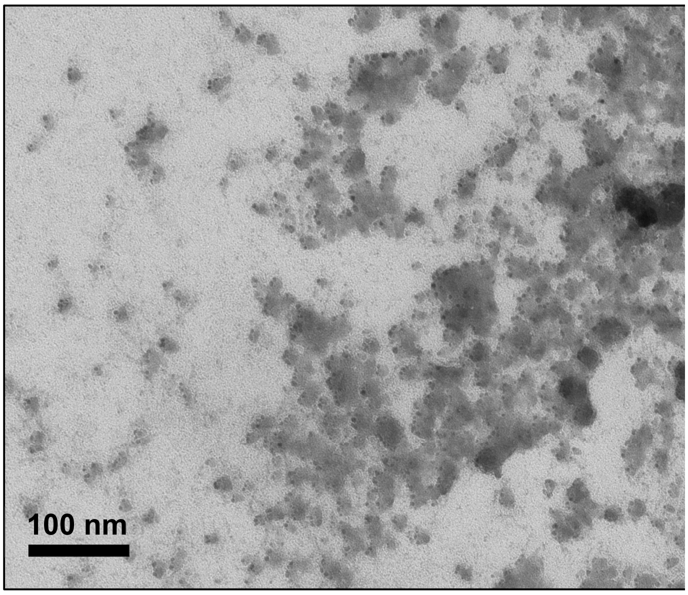

(a)

Critical Quality Attribute	
Particle size (TEM)	23.8 ± 3.4 nm
Dispersity index	0.763
Zeta potential (in PBS)	2.303 ± 1.54 mV
RNA concentration (before encapsulation)	2 nM
RNA concentration (after encapsulation)	1.92 ± 0.24 nM
**Encapsulation efficiency**	**~96%**

(b)

Figure S1. Physicochemical characterization of the nanoliposomes used in this study. (**a**) transmission electron micrograph (TEM) of nanoliposome population. Particle agglomeration is moderate because liposomes easily disperse when in aqueous suspension. (**b**) quantitative data collected from TEM, dynamic light scattering (DLS), and fluorescence spectroscopy. Data was collected in triplicate over four different experiments (n=12). For particle size analysis, only individual liposomes were measured.
